# Supplementary material for: Disentangling signal and noise in neural responses through generative modeling
Source: PLoS Comput Biol. 2025 Jul 21;21(7):e1012092. doi: 10.1371/journal.pcbi.1012092 (PMC12289057; doi:10.1371/journal.pcbi.1012092)
Supplement: S1 Fig — (PDF) [file pcbi.1012092.s001.pdf]

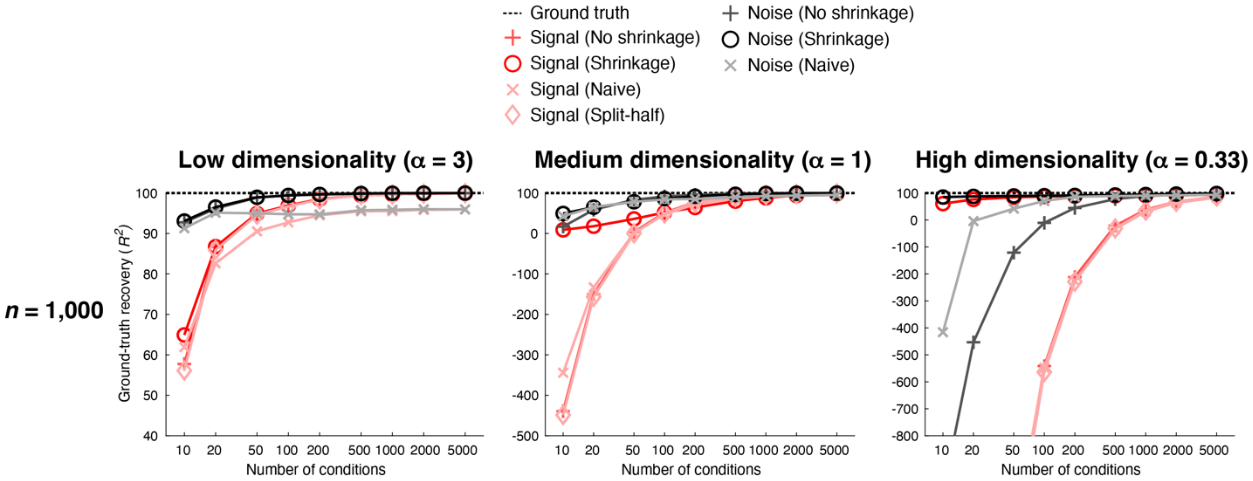

S1 Fig. Ground-truth recovery of covariance for a very large number of units. Same format as **Fig 4C**. Notice that compared to **Fig 4C** (where *n* = 10 and *n* = 50), larger numbers of conditions are necessary for accurate ground-truth recovery in the scenarios depicted in this figure (where *n* = 1,000). This is most pronounced in the case of high dimensionality (right) and methods that do not incorporate shrinkage (e.g. Signal (Split-half)). Nonetheless, we see that the GSN method (with shrinkage) is able to achieve high performance with only a moderate number of conditions (~500) in all three scenarios.
