# Supplementary material for: Disentangling signal and noise in neural responses through generative modeling
Source: PLoS Comput Biol. 2025 Jul 21;21(7):e1012092. doi: 10.1371/journal.pcbi.1012092 (PMC12289057; doi:10.1371/journal.pcbi.1012092)
Supplement: S2 Fig — (PDF) [file pcbi.1012092.s002.pdf]

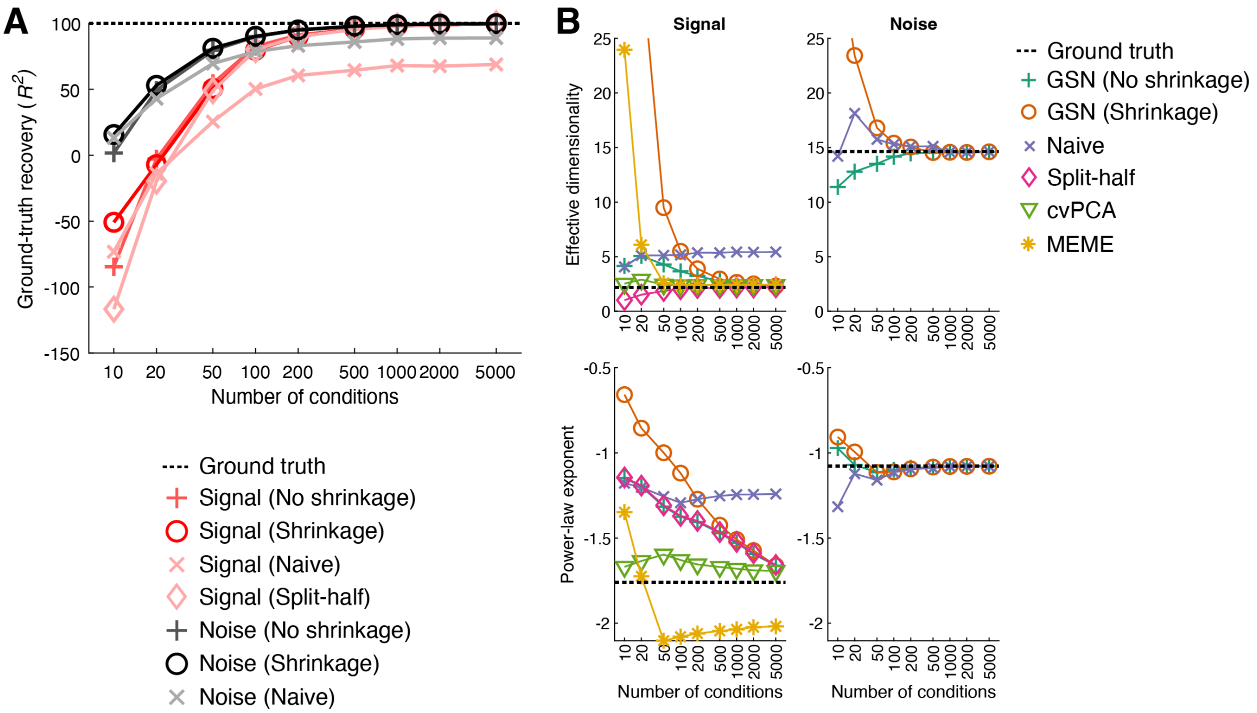

S2 Fig. Simulations for empirically derived signal and noise covariance. Here, we show simulation results for a scenario in which we take the ground-truth signal and noise covariance to be the GSN estimates of signal and noise covariance obtained from FFA-1 as illustrated in **Fig 6** (code available at https://osf.io/3yvtg). *A*, Same format as **Fig 4C**. Results are similar to those found in **Fig 4C**. *B*, Same format as **Fig 5**. The results for effective dimensionality (ED) look similar to those found in **Fig 5**. However, the results for power-law exponent look different. Specifically, the methods exhibit poor recovery of signal power-law exponent: each method either takes a very large amount of data to converge towards ground truth or has biases that do not resolve with additional data. One potential explanation is that the ground-truth signal covariance in this scenario is not exactly a line in log-log space (i.e. a power-law function), whereas all of the scenarios shown in **Fig 5** are exactly linear in log-log space. Hence, recovery may be especially difficult to achieve for the current scenario. Arguably, ED is a more appropriate metric for the evaluation of methods here, as it makes minimal assumptions about the structure of the eigenspectrum. Also, note that for sake of consistency with the other simulations, the MEME method was run assuming an unbroken power-law function; in theory, the MEME method could be run assuming a broken power-law function, which might help better match the ground-truth signal eigenspectrum and improve results.
