# Supplementary material for: Disentangling signal and noise in neural responses through generative modeling
Source: PLoS Comput Biol. 2025 Jul 21;21(7):e1012092. doi: 10.1371/journal.pcbi.1012092 (PMC12289057; doi:10.1371/journal.pcbi.1012092)
Supplement: S3 Fig — (PDF) [file pcbi.1012092.s003.pdf]

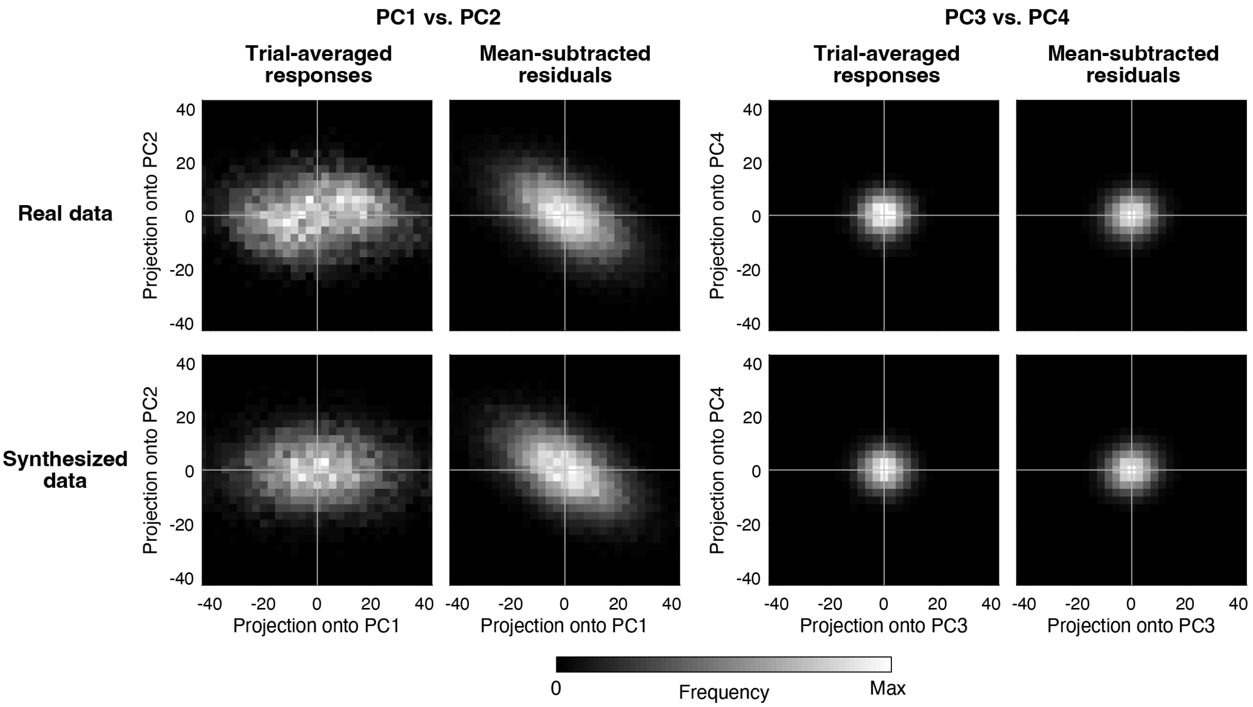

S3 Fig. Assessment of data distributions. As an instructive exercise, we take the empirical brain data from FFA-1 illustrated in **Fig 6** and perform an inspection of the signal and noise components of the data (code available at https://osf.io/yxrsp). We inspect two different distributions. One is the distribution of trial-averaged responses. Since trial averaging reduces noise, inspecting trial-averaged responses helps us assess properties of the signal. The second is the distribution of mean-subtracted residuals (in which trial-averaged responses have been removed). This allows us to focus our assessment on properties of the noise. We compute the first several principal components (PCs) of the covariance of the trial-averaged responses and then visualize the two distributions of interest in the low-dimensional space defined by these PCs. We also generate, for comparison, a synthesized dataset based on the parameters of the GSN model as fit to the empirical data. In order to generate responses for this synthesized dataset, we assume that both the signal and noise are Gaussian-distributed. We visualize the synthesized data in exactly the same manner as the real data (including using the same low-dimensional space). Examining the distributions associated with the real data (top row), we see that both the distribution of trial-averaged responses and the distribution of mean-subtracted residuals are Gaussian-like in their shape. We also see that the structure of the mean-subtracted residuals differs from that of the trial-averaged responses (top row, compare first and second images). This indicates that the noise structure is not identical to the signal structure, consistent with the inspections in **Fig 6A**. Next, we compare the distributions associated with the real data (top row) with those obtained from the synthesized data (bottom row). The distributions obtained from the synthesized data look very similar to those from the real data, suggesting that both the signal and the noise in the real data have Gaussian-like distributions and that the generative model learned by GSN accurately characterizes the real data.
