# Supplementary material for: Disentangling signal and noise in neural responses through generative modeling
Source: PLoS Comput Biol. 2025 Jul 21;21(7):e1012092. doi: 10.1371/journal.pcbi.1012092 (PMC12289057; doi:10.1371/journal.pcbi.1012092)
Supplement: S4 Fig — (PDF) [file pcbi.1012092.s004.pdf]

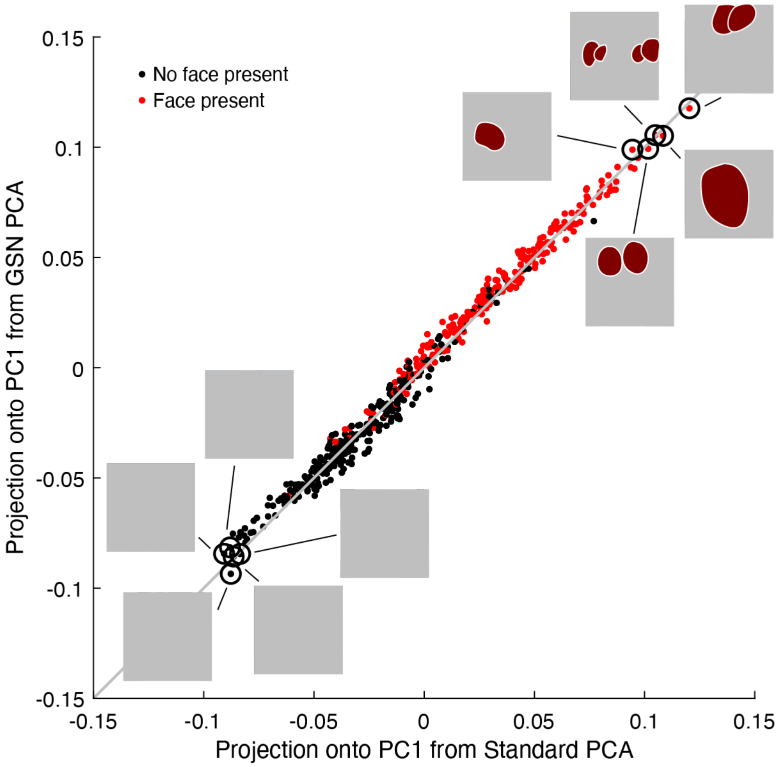

S4 Fig. Inspection of stimuli in PCA results. Here we visually inspect stimulus images from the PCA analysis (code available at https://osf.io/f34bc). The projections of responses in FFA-1 to the common 515 images onto PC1 (see **Fig 7C**) were unit-length-normalized, averaged across participants, and then unit-length-normalized again. This figure compares the results obtained using Standard PCA (*x*-axis) against the results obtained using GSN PCA (*y*-axis). Red dots indicate images that were judged by human raters to have at least one prominent face present; black dots indicate all other images. (The human raters were blind to the results in this paper.) The actual images corresponding to the highest five and lowest five projection values (based on the average of the results of the two methods) are shown. Dark red shapes indicate locations of faces present in the images (the actual images are not shown due to copyright reasons). The presence of faces appears to be the dominant factor governing the response projections.
