# Supplementary material for: Disentangling signal and noise in neural responses through generative modeling
Source: PLoS Comput Biol. 2025 Jul 21;21(7):e1012092. doi: 10.1371/journal.pcbi.1012092 (PMC12289057; doi:10.1371/journal.pcbi.1012092)
Supplement: S5 Fig — (PDF) [file pcbi.1012092.s005.pdf]

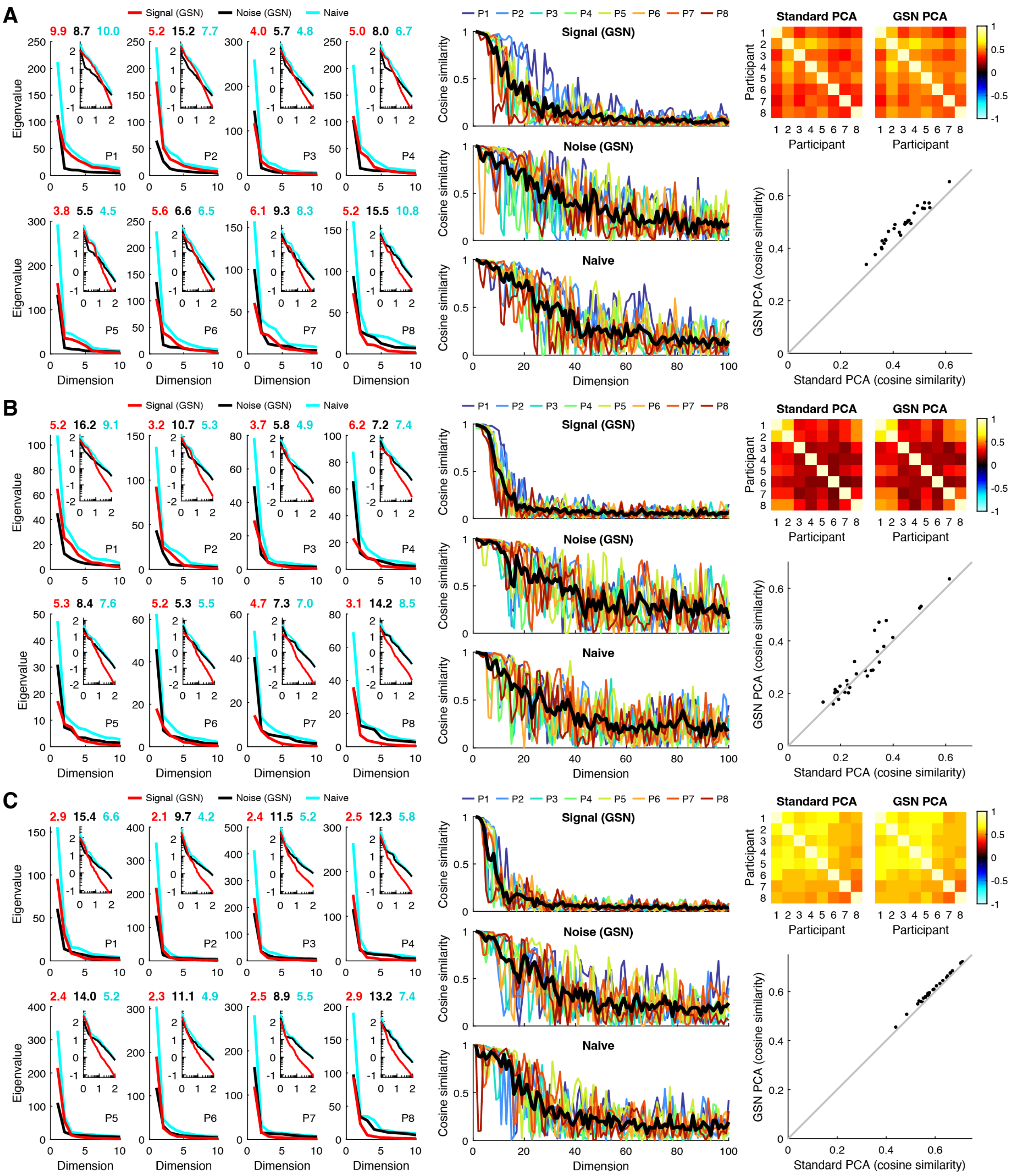

S5 Fig. PCA results for additional brain regions. Here we show results of the PCA analysis for additional brain regions (code available at https://osf.io/f34bc). The format is the same as used in **Fig 7**. *A–C*, Results for V1, hV4, and PPA, respectively. The main findings observed for FFA-1 in **Fig 7** replicate for these additional regions, including lower dimensionality for the signal compared to the noise, high within-participant reliability of the first several signal PCs and noise PCs, and higher across-participant consistency of trial-averaged response projections onto PC1 for GSN PCA than for standard PCA. Compared to FFA-1, the increase in across-participant consistency is more variable in hV4 and is relatively small (but reliable)

in PPA. One possible source of these region-wise differences may be differences in the degree to which signal covariance structure and noise covariance structure are aligned in different brain regions. For example, if noise covariance tends to align with signal covariance, then noise may have less of a corrupting influence on the estimation of signal PCs compared to when noise covariance is orthogonal to signal covariance.
