# Supplementary material for: Disentangling signal and noise in neural responses through generative modeling
Source: PLoS Comput Biol. 2025 Jul 21;21(7):e1012092. doi: 10.1371/journal.pcbi.1012092 (PMC12289057; doi:10.1371/journal.pcbi.1012092)
Supplement: S1 Appendix — (PDF) [file pcbi.1012092.s006.pdf]

S1 Appendix: GSN estimation of signal and noise covariance

Problem setting

As described in the main text, GSN calculates two covariance estimates from the data: $\hat{\Sigma}_{noiseORIG}$ and $\hat{\Sigma}_{data[t]}$. The former is an estimate of the noise covariance based on the trial-to-trial variability around the mean response to each condition (see Step 2). The latter is an estimate of the data covariance based on the data after averaging across t trials (see Step 3).

These two covariance estimates reflect unknown covariance matrices Σ_{signal} and Σ_{noise} such that $\hat{\Sigma}_{noiseORIG}$ is a noisy version of Σ_{noise} based on $c(t-1)$ samples and $\hat{\Sigma}_{data[t]}$ is a noisy version of $\Sigma_{data[t]} = \Sigma_{signal} + \Sigma_{noise}/t$ based on $c-1$ samples.

We wish to determine estimates $\hat{\Sigma}_{signal}$ and $\hat{\Sigma}_{noise}$ under the constraint that these estimates are positive semi-definite matrices. To do so, we define the following loss that quantifies errors from the data-derived covariances scaled by the number of samples they are based on:

$$L(\hat{\Sigma}_{signal}, \hat{\Sigma}_{noise}) = c(t-1)\left\|\hat{\Sigma}_{noiseORIG} - \hat{\Sigma}_{noise}\right\|_2^2 + (c-1)\left\|(\hat{\Sigma}_{data[t]} - \hat{\Sigma}_{noise}/t) - \hat{\Sigma}_{signal}\right\|_2^2$$

where $\| \|_2$ indicates the Frobenius norm. Intuitively, the noise estimate $\hat{\Sigma}_{noise}$ is allowed to deviate to some degree from the data-derived $\hat{\Sigma}_{noiseORIG}$, and the signal estimate $\hat{\Sigma}_{signal}$ is allowed to deviate to some degree from the subtraction-based estimate of the signal covariance $\hat{\Sigma}_{data[t]} - \hat{\Sigma}_{noise}/t$.

Notice $\hat{\Sigma}_{noiseORIG}$ is positive semi-definite, as it is a covariance matrix computed from data. If $\hat{\Sigma}_{data[t]} - \hat{\Sigma}_{noise}/t$ is also positive semi-definite, setting $\hat{\Sigma}_{noise} = \hat{\Sigma}_{noiseORIG}$ and $\hat{\Sigma}_{signal} = \hat{\Sigma}_{data[t]} - \hat{\Sigma}_{noise}/t$ is the optimal solution for the problem (since the loss equals zero). If not, we can solve the optimization problem using the method described below.

Solution

To solve the optimization problem in the general case, we note that it is a convex optimization problem, as it is a sum of squares and the cone of semi-definite matrices is a convex set [1]. Thus, this problem has a single optimum. For solving this problem efficiently, we split the problem into optimizing $\hat{\Sigma}_{signal}$ and $\hat{\Sigma}_{noise}$ separately, as we can compute an analytic solution for each matrix if the other is fixed. Since each of these separate optimizations is guaranteed to improve the loss, this approach is guaranteed to converge.

Lemma: solution pattern

Consider the following problem. Given B, find A that minimizes $\|B - A\|_2^2$ (or equivalently $\|A - B\|_2^2$) subject to the constraint that A is positive semi-definite. We can solve this problem as $A = \mathrm{PSD}(B)$ where $\mathrm{PSD}()$ is the method for finding the nearest positive semi-definite matrix described in the main text. We will use this solution pattern in solving the individual optimizations for $\hat{\Sigma}_{signal}$ and $\hat{\Sigma}_{noise}$.

Optimizing $\hat{\Sigma}_{signal}$

Since $c(t-1)\left\|\hat{\Sigma}_{noiseORIG} - \hat{\Sigma}_{noise}\right\|_2^2$ is independent of $\hat{\Sigma}_{signal}$, we are left with minimizing

$$(c-1)\left\|\left(\hat{\Sigma}_{data[t]}-\hat{\Sigma}_{noise}/t\right)-\hat{\Sigma}_{signal}\right\|_2^2$$

subject to $\hat{\Sigma}_{signal}$ being positive semi-definite. To do this, we use our solution pattern where $A=\hat{\Sigma}_{signal}$ and $B=\hat{\Sigma}_{data[t]}-\hat{\Sigma}_{noise}/t$.

Optimizing $\hat{\Sigma}_{noise}$

In this case, we apply a quadratic extension to turn the sum of squares into a single one:

$$L\left(\hat{\Sigma}_{signal},\hat{\Sigma}_{noise}\right)=\sum_{ij}\left[c(t-1)\left(\hat{\Sigma}_{noiseORIG}^{(ij)}-\hat{\Sigma}_{noise}^{(ij)}\right)^2+(c-1)\left(\hat{\Sigma}_{data[t]}^{(ij)}-\hat{\Sigma}_{signal}^{(ij)}-\frac{\hat{\Sigma}_{noise}^{(ij)}}{t}\right)^2\right]$$

$$=\sum_{ij}\left[c(t-1)\left(\left(\hat{\Sigma}_{noiseORIG}^{(ij)}\right)^2-2\hat{\Sigma}_{noiseORIG}^{(ij)}\hat{\Sigma}_{noise}^{(ij)}+\left(\hat{\Sigma}_{noise}^{(ij)}\right)^2\right)\right.$$
$$\left.+\frac{c-1}{t^2}\left(\left(t^2\left(\hat{\Sigma}_{data[t]}^{(ij)}-\hat{\Sigma}_{signal}^{(ij)}\right)^2\right)-2t\left(\hat{\Sigma}_{data[t]}^{(ij)}-\hat{\Sigma}_{signal}^{(ij)}\right)\hat{\Sigma}_{noise}^{(ij)}+\left(\hat{\Sigma}_{noise}^{(ij)}\right)^2\right)\right]$$

$$=\sum_{ij}\left[\frac{ct^2(t-1)+c-1}{t^2}\left(\hat{\Sigma}_{noise}^{(ij)}\right)^2-2\hat{\Sigma}_{noise}^{(ij)}\left(c(t-1)\hat{\Sigma}_{noiseORIG}^{(ij)}+\frac{c-1}{t}\left(\hat{\Sigma}_{data[t]}^{(ij)}-\hat{\Sigma}_{signal}^{(ij)}\right)\right)\right]+C_0$$

where C_0 is a term that is independent of $\hat{\Sigma}_{noise}$. Simplifying, we obtain:

$$L\left(\hat{\Sigma}_{signal},\hat{\Sigma}_{noise}\right)\propto\sum_{ij}\left[\hat{\Sigma}_{noise}^{(ij)}-\frac{ct^2(t-1)}{ct^2(t-1)+c-1}\hat{\Sigma}_{noiseORIG}^{(ij)}-\frac{c-1}{ct^2(t-1)+c-1}t\left(\hat{\Sigma}_{data[t]}^{(ij)}-\hat{\Sigma}_{signal}^{(ij)}\right)\right]^2+C_1$$

where C_1 is a term that is independent of $\hat{\Sigma}_{noise}$. To minimize this loss, we use our solution pattern where $A=\hat{\Sigma}_{noise}$ and $B=\frac{ct^2(t-1)}{ct^2(t-1)+c-1}\hat{\Sigma}_{noiseORIG}+\frac{c-1}{ct^2(t-1)+c-1}t\left(\hat{\Sigma}_{data[t]}-\hat{\Sigma}_{signal}\right)$.

Notice that the calculation of B is a weighted average of two possible estimates of the noise covariance. The first estimate, $\hat{\Sigma}_{noiseORIG}$, reflects the covariance of mean-subtracted residuals, while the second estimate, $t\left(\hat{\Sigma}_{data[t]}-\hat{\Sigma}_{signal}\right)$, reflects the subtraction of the signal distribution from the data distribution. The weights in the weighted average reflect the amount of data that inform each of the two estimates.

Algorithm

The overall algorithm for optimizing signal and noise covariance estimates is described in the main text. Holding $\hat{\Sigma}_{noise}$ fixed, the algorithm optimizes $\hat{\Sigma}_{signal}$ in Step 6.1. Holding $\hat{\Sigma}_{signal}$ fixed, the algorithm optimizes $\hat{\Sigma}_{noise}$ in Step 6.2. This process of biconvex optimization is iterated until convergence.

Proof that projection reduces error

We claim in the main text that projection of a given covariance estimate onto the positive semi-definite cone always reduces the error of the estimate. Here we provide a simple proof of this claim.

Definitions: For this proof, let Σ be the true *n*-dimensional covariance which lies within the convex cone of positive semi-definite matrices $C\subset\mathbb{R}^{n\times n}$. We assume the original covariance estimate $\hat{\Sigma}\notin C$.

Theorem: Under these conditions, the squared error of the projection onto the positive semi-definite cone $\mathrm{PSD}(\hat{\Sigma})$ is smaller than the squared error of the original estimate, i.e.:

$$\left\|\Sigma-\mathrm{PSD}(\hat{\Sigma})\right\|_2^2<\left\|\Sigma-\hat{\Sigma}\right\|_2^2$$

Proof: As C is convex, there is a tangent plane touching C at $\mathrm{PSD}(\hat{\Sigma})$ to which the vector from $\hat{\Sigma}$ to $\mathrm{PSD}(\hat{\Sigma})$ is orthogonal. All points in C are on the other side of this tangent plane compared to $\hat{\Sigma}$. The squared distance from $\hat{\Sigma}$ to Σ can be decomposed into the distance orthogonal to the tangent plane and the distance within the tangent plane. The distance within the plane is the same for $\hat{\Sigma}$ and $\mathrm{PSD}(\hat{\Sigma})$, and the distance orthogonal to the plane is smaller for $\mathrm{PSD}(\hat{\Sigma})$. Thus, the total distance for $\mathrm{PSD}(\hat{\Sigma})$ is indeed smaller than the total distance for $\hat{\Sigma}$. See **Fig S6.1** for a helpful illustration.

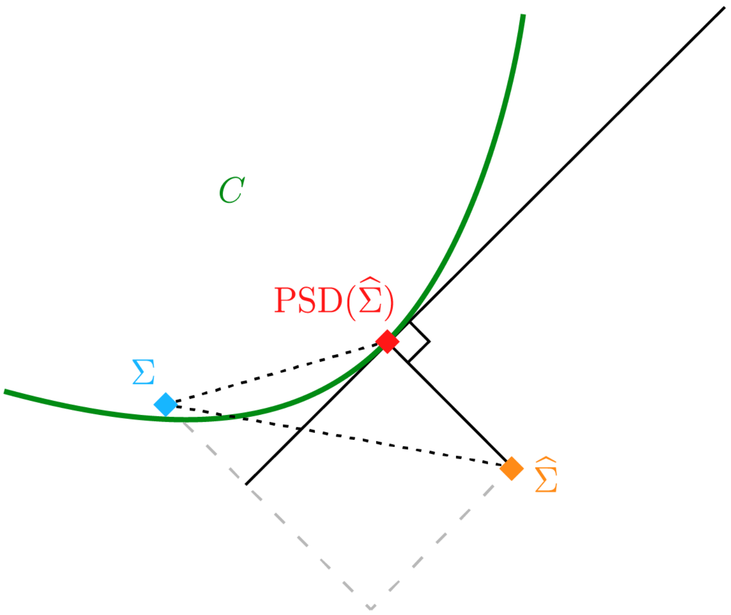

Fig S6.1. Illustration that projection onto the positive semi-definite cone reduces error.

Rationale for squared error

Our estimates are based on minimizing sum of squares, i.e., we minimize the squared difference between our estimates and the data-derived $\hat{\Sigma}_{noiseORIG}$ and $\hat{\Sigma}_{data[t]}$. Squared error is a common loss for the estimation of covariance matrices, and in particular, it is the loss optimized by the shrinkage method we employ for covariance estimation. Additionally, squared error is a convex loss function, which guarantees that our fitting procedure converges.

We note that our squared-error loss does not correspond to a log likelihood under some distributional assumption. Rather, it is merely a mathematically convenient way to express the trade-off between the two data-driven covariance estimates $\hat{\Sigma}_{noiseORIG}$ and $\hat{\Sigma}_{data[t]}$. Typical likelihood functions for covariance matrices imply larger variabilities for larger entries in the covariance matrix, but this is not the case for our squared-error loss.

In our squared-error loss, we weight the two errors (one for $\hat{\Sigma}_{noiseORIG}$, one for $\hat{\Sigma}_{data[t]}$) by the relevant degrees of freedom. This is a sensible approach that adapts to the specific numbers of conditions and trials used in a given experiment. We acknowledge that it may be possible to devise a more principled approach for determining the weighting. Nonetheless, note that the relative weighting of the errors does not change

asymptotic properties of the estimators as long as both weights are positive. For any chosen weighting, $\hat{\Sigma}_{noise}$ and $\hat{\Sigma}_{signal}$ are positive semi-definite and approximate the data-derived covariance estimates.

Improvement upon a simpler approach

A simple approach to ensuring that the signal and noise covariance estimates are positive semi-definite is to simply truncate the eigenspectrum of the signal covariance estimate (setting negative eigenvalues to zero). While this does ensure positive semi-definite covariance estimates, it is not as optimal as the full procedure that we propose. To illustrate why this is the case, we perform a small-scale simulation (**Fig S6.2**). Compared to simple truncation, our optimization procedure reduces the distance to the empirical data covariance matrix. In other words, it improves goodness-of-fit to the data.

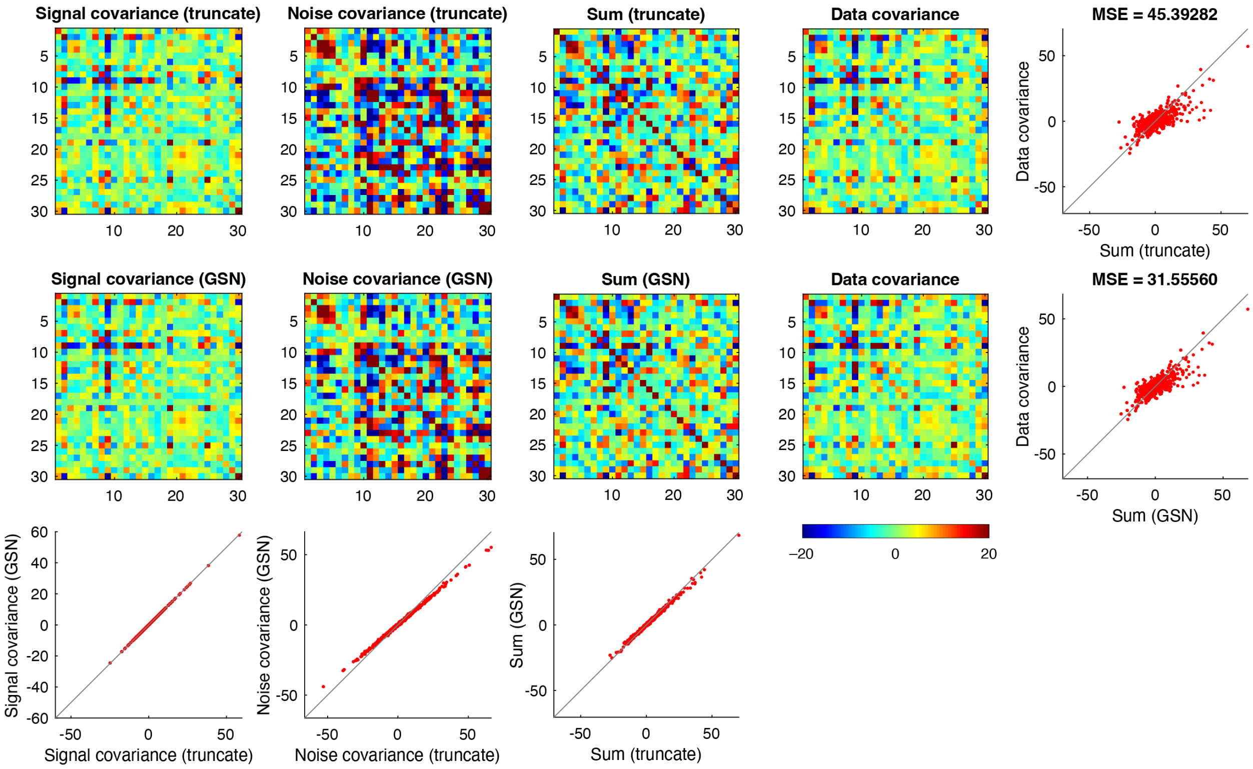

Fig S6.2. Comparison of GSN to simple truncation. Here we perform a simple simulation to illustrate the difference between a simple truncation method (in which the eigenspectrum of the signal covariance estimate is truncated, setting negative eigenvalues to zero) and GSN (code available at https://osf.io/sekjt). In the simulation, we generate random data for the case of 30 units × 5 conditions × 2 trials (signal drawn from a normal distribution with standard deviation 1; noise drawn from a normal distribution with standard deviation 5). The first row shows results obtained using the simple truncation method where noise covariance is estimated, data covariance is estimated, noise covariance is subtracted from data covariance yielding the signal covariance estimate, and then the eigenvalues of the signal covariance estimate are truncated. The third column shows the sum of the signal and noise covariance estimates. The fourth column shows the actual covariance of the trial-averaged data. The fifth column compares the sum to the actual covariance (with mean squared error indicated in the title). The second row shows results obtained using the GSN method (no shrinkage). Finally, the third row compares results obtained with the truncation method against those obtained with the GSN method for the signal covariance, noise covariance, and the sum. Notice that the results of the truncation method and GSN are highly similar but not identical. Also, notice that GSN yields a sum that is closer to the trial-averaged covariance. Hence, by iteratively adjusting the signal and noise covariance estimates, GSN is improving the goodness-of-fit to the data.

References

1. Boyd S, Vandenberghe L. Convex Optimization. Cambridge, England: Cambridge University Press; 2016.
