# Supplementary material for: Disentangling signal and noise in neural responses through generative modeling
Source: PLoS Comput Biol. 2025 Jul 21;21(7):e1012092. doi: 10.1371/journal.pcbi.1012092 (PMC12289057; doi:10.1371/journal.pcbi.1012092)
Supplement: S2 Appendix — (PDF) [file pcbi.1012092.s007.pdf]

S2 Appendix: Shrinkage-based covariance estimation

A core component of GSN is estimation of covariance (this is performed for the estimated noise covariance in Step 2 and the estimated data covariance in Step 3). However, in high-dimensional datasets involving a large number of units but only a limited number of samples (e.g. trials), the standard method of computing sample covariance may yield inaccurate estimates of covariance. To improve accuracy of covariance estimation, GSN incorporates shrinkage [1,2] of off-diagonal elements of covariance matrices towards zero. This reflects the prior that units are generally expected to be uncorrelated. The specific amount of shrinkage is tailored to optimally match the data using a cross-validation procedure in which likelihoods are evaluated on held-out data (see *Methods*).

We tested our shrinkage-based method for covariance estimation. We performed a set of simulations in which we assessed, as a function of the number of samples, how well the shrinkage method recovers a ground-truth covariance, compared to the standard method in which shrinkage is omitted (**Fig S7.1**). In one set of simulations, we used a ground-truth covariance equal to the identity matrix, corresponding to a scenario of uncorrelated units (panel A). In a second set of simulations, we re-used the previous ground-truth covariance but introduced positive correlations (r = 0.5) amongst the first five units (panel B). For additional comparison, in a third set of simulations, we used a ground-truth covariance equal to the covariance of a fixed set of random numbers drawn from the standard normal distribution (20 observations, 10 variables) (panel C).

The results show that the shrinkage method works well. In each scenario, the introduction of shrinkage improves ground-truth covariance recovery and this occurs regardless of the number of samples (panels A–C, lower left). Note that the size of the improvement varies across scenarios, with larger improvements when the ground truth is consistent with the prior of uncorrelated units (e.g. panel A) than when this is less the case (e.g. panel C). This makes sense: the cross-validation procedure should, in theory, correctly determine that shrinkage should be applied more strongly in situations where the underlying ground-truth covariance involves uncorrelated variables. Indeed, if we examine cross-validation results across different shrinkage levels, we see that in the scenario of uncorrelated variables, the shrinkage fractions yielding the highest likelihood on held-out data are close to 0, indicating large amounts of shrinkage (panel A, vertical red line in rightmost column), whereas in scenarios of correlated variables, the optimal shrinkage levels are closer to 1, indicating small amounts of shrinkage (panels B–C, vertical red lines in rightmost column).

The simulations also reveal insights into how ground-truth recovery performance varies as a function of the amount of data. As the number of samples increases, the induced shrinkage becomes weaker (compare 5 samples to 100 samples in panel C). This makes sense because at small sample sizes, the unregularized (non-shrunken) covariance is so inaccurate that inducing heavy bias improves the estimate. Furthermore, we see that as the number of samples increases, the difference in results between the shrinkage method and the standard method becomes smaller. Thus, shrinkage provides the most benefit when the amount of available data is small. It is important to keep in mind, however, that the covariance estimates produced by shrinkage are by no means perfect and that they contain bias. This can be seen intuitively by visually comparing the shrinkage-based covariance estimates at low number of samples to the ground-truth covariance. While shrinkage increases the overall similarity of covariance estimates to the underlying ground-truth covariance, it does so at the expense of biasing the magnitudes of off-diagonal elements towards zero. The introduction of bias is not necessarily a problem per se, as it depends on the goals of the researcher.

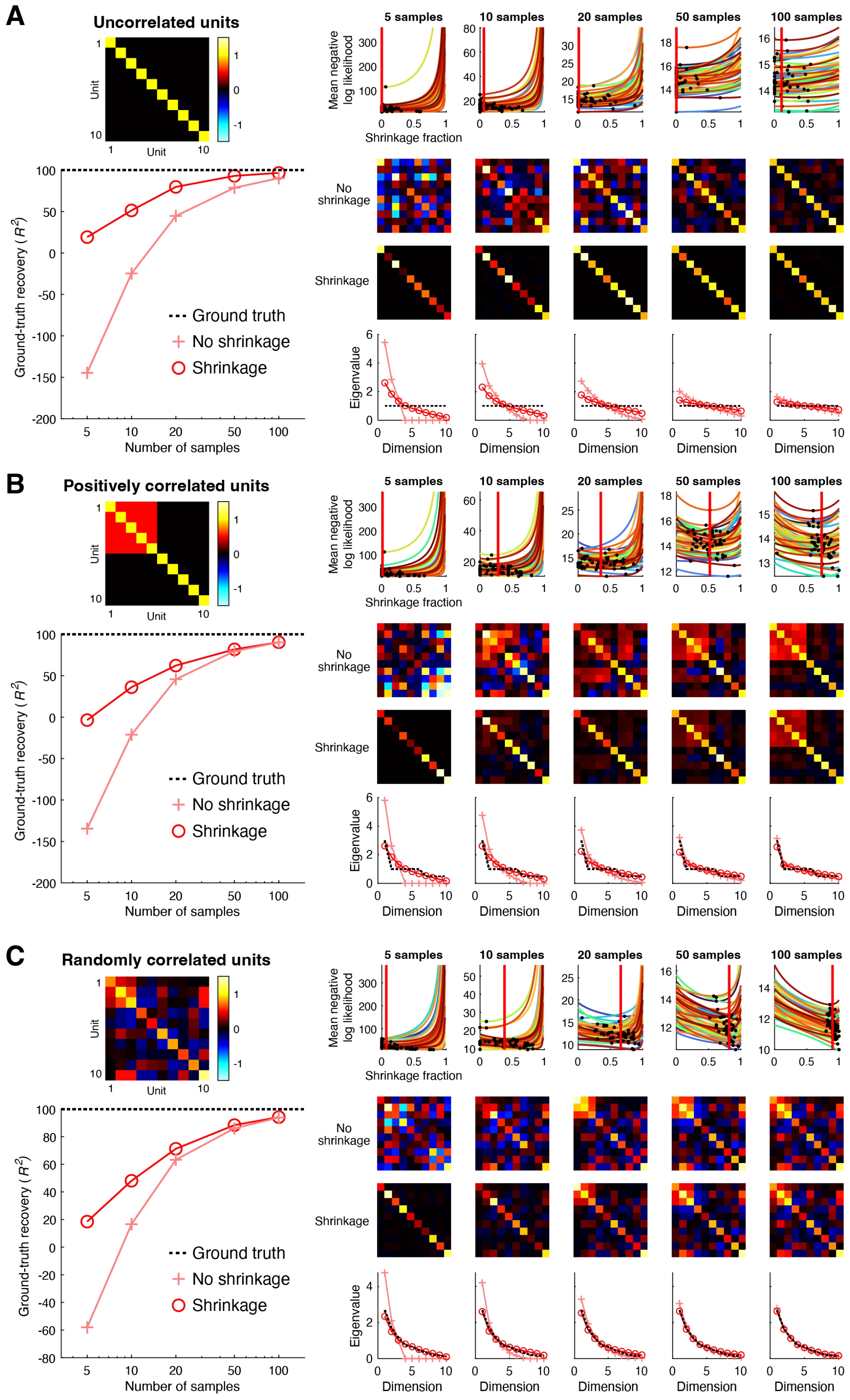
A
Uncorrelated units
Unit
Ground-truth recovery (R^2)
Number of samples
Ground truth
No shrinkage
Shrinkage
5 samples
10 samples
20 samples
50 samples
100 samples
Mean negative log likelihood
Shrinkage fraction
No shrinkage
Shrinkage
Eigenvalue
Dimension
B
Positively correlated units
C
Randomly correlated units

Fig S7.1. Shrinkage-based covariance estimation. Here we show results of simulations that assess the performance of the shrinkage-based method we use for covariance estimation (code available at https://osf.io/yr3vx). Panels A–C depict three different scenarios. Each scenario involves 10 units whose responses are distributed according to a ground-truth multivariate Gaussian (whose covariance is shown at the upper left). We vary the number of samples (e.g., trials, conditions) drawn from the distribution, performing 50 simulations for each number of samples. In each simulation, we estimate covariance from the samples using two different methods. One method ('No shrinkage') is to simply compute the sample covariance with Bessel's correction. The second method ('Shrinkage') involves additionally shrinking the off-diagonal elements of the sample covariance, using cross-validation to determine the optimal shrinkage level. In each panel, the ground-truth covariance is shown at the upper left. Cross-validation results for different numbers of samples are shown at the upper right, where colored lines indicate different simulations, black dots indicate the minimum negative log likelihood achieved, and the vertical red line indicates the median selected shrinkage level across simulations. Below each cross-validation plot, covariance estimates from one simulation are shown (we choose the simulation in which the selected shrinkage level is closest to the median). At the bottom are plots of the eigenspectra (mean across simulations) produced by the two methods (red and pink lines) as well as the ground-truth eigenspectrum (black dotted line). Finally, the ground-truth recovery performance quantified using coefficient of determination (R^2) is shown at the lower left (mean across simulations).

A clear benefit of the bias induced by shrinkage can be seen in the eigenspectra of the covariance estimates (panels A–C, bottom right). Even though the sample covariance provides an unbiased estimate of covariance, it produces biased eigenspectra that are lower in dimensionality than the ground-truth eigenspectra (see steep fall-off of the eigenspectra in the case of 5 samples). In other words, the sample covariance tends to underestimate the true dimensionality of the data. Shrinkage, to an extent, alleviates this issue, as it increases dimensionality (eigenvalues become more spread out) and produces eigenspectra that more closely resemble the ground-truth eigenspectra. These results are consistent with prior results from the literature (see Fig 1 in [2]).

References

1. Ledoit O, Wolf M. A well-conditioned estimator for large-dimensional covariance matrices. J Multivar Anal. 2004;88: 365–411.
2. Schäfer J, Strimmer K. A shrinkage approach to large-scale covariance matrix estimation and implications for functional genomics. Stat Appl Genet Mol Biol. 2005;4: Article32.
